# Involvement of a host Cathepsin L in symbiont‐induced cell death

**DOI:** 10.1002/mbo3.632

**Published:** 2018-04-24

**Authors:** Suzanne M. Peyer, Natacha Kremer, Margaret J. McFall‐Ngai

**Affiliations:** ^1^ School of Medicine and Public Health Department of Medical Microbiology and Immunology University of Wisconsin Madison WI USA; ^2^ McPherson Eye Research Institute University of Wisconsin Madison WI USA; ^3^ Present address: Laboratoire de Biométrie et Biologie Evolutive UMR CNRS 5558 Université Lyon 1 Université de Lyon Villeurbanne France; ^4^ Present address: Pacific Biosciences Research Center University of Hawai'i at Manoa Honolulu HI USA

**Keywords:** apoptosis, cathepsin, cell death, cysteine protease, development, morphogenesis, symbiosis

## Abstract

The *cathepsin L* gene of the host squid, *Euprymna scolopes*, is upregulated during the first hours of colonization by the symbiont *Vibrio fischeri*. At this time, the symbiotic organ begins cell death‐mediated morphogenesis in tissues functional only at the onset of symbiosis. The goal of this study was to determine whether Cathepsin L, a cysteine protease associated with apoptosis in other animals, plays a critical role in symbiont‐induced cell death in the host squid. Sequence analysis and biochemical characterization demonstrated that the protein has key residues and domains essential for Cathepsin L function and that it is active within the pH range typical of these proteases. With in situ hybridization and immunocytochemistry, we localized the transcript and protein, respectively, to cells interacting with *V. fischeri*. Activity of the protein occurred along the path of symbiont colonization. A specific Cathepsin L, nonspecific cysteine protease, and caspase inhibitor each independently attenuated activity and cell death to varying degrees. In addition, a specific antibody decreased cell death by ~50%. Together these data provide evidence that Cathepsin L is a critical component in the symbiont‐induced cell death that transforms the host tissues from a colonization morphology to one that promotes the mature association.

## 1 INTRODUCTION

Cathepsins are a family of lysosomal proteases, categorized as serine (A, G), cysteine (B, C, F, H, K, L, O, S, V, X, W), or aspartic (D, E) depending on the amino acid essential for activity (Conus & Simon, 2008; Niu, Jin, Wang, Feng, & Li, 2013). Degrading materials identified by the cell as unusable or foreign, cathepsins have been implicated in mediating such varied processes as development and immunity (Fonović & Turk, 2014; Repnik, Stoka, Turk, & Turk, 2012). A key signature of these proteases is their stability under the acidic conditions of the lysosomes with limited activity at elevated pH (Arockiaraj et al., 2013; Conus & Simon, 2008; Fonović & Turk, 2014).

In development, cathepsins serve a role in the death of old and genesis of new cells. Along with the caspase and calpain proteases, cathepsins are involved in cell death, including apoptosis (Conus & Simon, 2008; Repnik et al., 2012). For instance, some cathepsins can become activated (Baumgartner et al., 2007), released into the cytosol (Chwieralski, Welte, & Bühling, 2006), and degrade antiapoptotic proteins (Repnik et al., 2012). Other cathepsins process granzyme B molecules that enter and destroy target cells (Repnik et al., 2012) or cleave procaspase, increasing activity of the mature form (Yang, Yin, & Xu, 2017). Yet other cathepsins are secreted out of a cell and help digest extracellular matrices (Fonović & Turk, 2014). Such activities shape tissues. For example, *cathepsin B, D*, and *L* transcripts are regulated in the interdigital development of chick and duck embryos, a process that involves dramatic cell‐death‐associated restructuring (Zuzarte‐Luis, Montero, Kawakami, Izpisua‐Belmonte, & Hurle, 2007). In contrast, in the marine mussel *Mytilus edulis*, Cathepsin L activity is higher during oocyte development and may be involved in yolk formation (Gabbott & Peek, 1991) rather than cell death.

Cathepsins also play a role in both beneficial and pathogenic animal–microbe interactions (Flannagan, Cosío, & Grinstein, 2009). In a recent study, Cathepsin K was identified as protective against chronic inflammation in the gut of mammals (Sina et al., 2013). Regulation of cathepsins also occurs in response to infections. Of particular interest is Cathepsin L, whose transcript is upregulated in response to *Vibrio* spp. in several mollusks, including the razor clam *Sinonovacula constricta* (Niu et al., 2013), the soft‐shell clam *Mya arenaria* (Araya et al., 2010), and the pearl oyster *Pinctada fucata* (Ma et al., 2010). In the freshwater prawn *Macrobrachium rosenbergii*, the *cathepsin L* transcript is upregulated in response to bacterial (*Vibrio harveyi* and *Aeromonas hydrophila*) and viral (*M*. *rosenbergii* nodovirus or white spot syndrome baculovirus) infections (Arockiaraj et al., 2013). Lipopolysaccharide (LPS) is a cue known to upregulate *cathepsin L* in the red crayfish *Procambarus clarkii* (Dai, Chu, Yu, & Li, 2017). Interestingly, caspases are also receptors for LPS in other systems (Shi et al., 2014).

**Figure 1:**
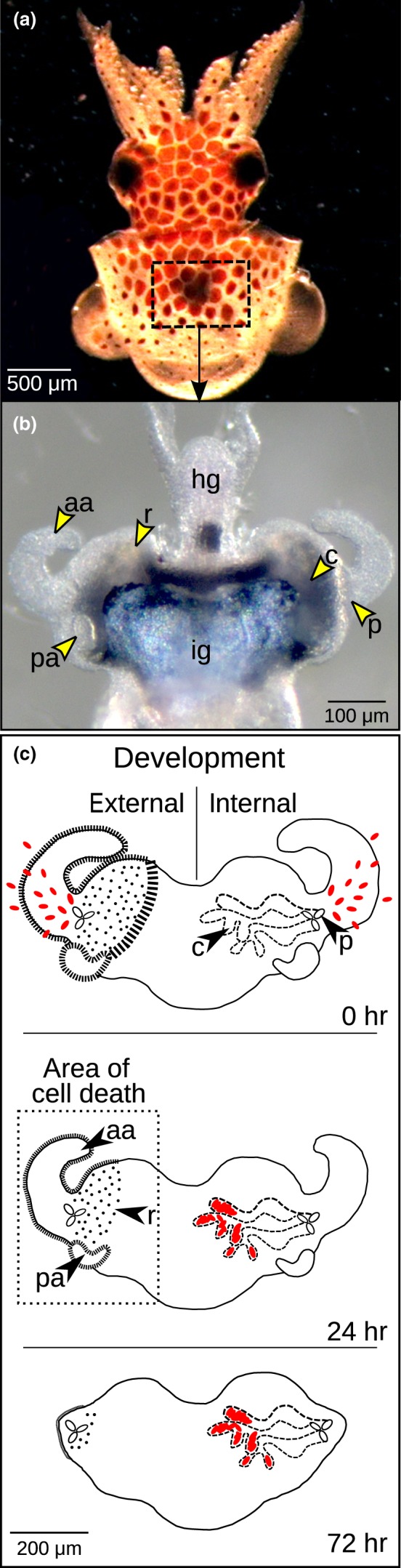
The *Euprymna scolopes*‐*Vibrio fischeri* system. (a) Juvenile *E. scolopes* with the region of the light organ outlined by the box. (b) Juvenile light organ, derived from digestive tissues, is surrounded by the ink gland (ig) and attached to the hindgut (hg). The remaining morphological features of the light organ are also illustrated and described in the following. (c) Symbiont‐induced development of the juvenile light organ. Over the first hours of interaction, selection of *V. fischeri* (red ovals) occurs on the ciliated “external” surface. The bacteria migrate through three pores (p) on either side of the organ and ultimately reside within the “internal” crypts (c). The superficial tissues that undergo cell death include the anterior appendages (aa), posterior appendages (pa), and the ciliated ridges (r), a developmental process that completes within ~96 hr. Morphogenesis continues for several additional days to weeks with the crypt spaces developing into a central core and the three pores forming a single pore

The Hawaiian bobtail squid *Euprymna scolopes* and its microbial symbiont *V*. *fischeri* is a model system for studying host–microbe interactions. The partnership is established shortly after the host hatches (Figure 1a), with the symbiont, harvested from the surrounding seawater, colonizing a specialized “light organ” (Figure 1b) within hours of first contact. Initially, the *V*. *fischeri* cells interact with the ciliated appendages of the light organ, which are superficial epithelial tissues present only in the juvenile host (Figure 1b and c). The symbiont cells then attach to the beating cilia (Altura et al., 2013) and aggregate at the pores on the organ surface before traveling to their final destination, which is a set of epithelium‐lined crypts deep in the tissues (Figure 1c; Yip, Geszvain, Deloney‐Marino, & Visick, 2006). The juvenile host is exposed to many bacterial species in the seawater, and all gram‐negative bacteria examined attach to the cilia (Nyholm, Deplancke, Gaskins, Apicella, & McFall‐Ngai, 2002). However, when *V*. *fischeri* is present, it becomes a competitive dominant in the aggregates and only *V*. *fischeri* successfully passes through the ducts to colonize the crypts. During these first few hours of infection, the *cathepsin L* transcript is upregulated in the host (Kremer et al., 2013).

Once the symbiont has entered the crypts, the light organ transforms through a cell‐death‐dependent morphogenic process (Figure 1c) that results in the loss of the complex ciliated fields that facilitate symbiont colonization of the host. Although complemented by symbiont light production, the primary morphogens include the *V. fischeri* cell‐envelope molecules lipid A and tracheal cytotoxin (TCT), derivatives of lipopolysaccharide (LPS) and peptidoglycan (Foster, Apicella, & McFall‐Ngai, 2000; Koropatnick, Kimbell, & McFall‐Ngai, 2007; Koropatnick et al., 2004), respectively. These “microbe‐associated molecular patterns,” or MAMPs, provide signals whereby animals recognize microbes in both beneficial and pathogenic symbioses (Nyholm & Graf, 2012). Once cell death is triggered by these symbiont cues, host hemocytes migrate into the anterior appendages of the light organ (Figure 1b and c) (Koropatnick et al., 2007) and metalloproteinases break down the extracellular matrices in the targeted degenerating tissues (Koropatnick, Goodson, Heath‐Heckman, & McFall‐Ngai, 2014). Whether Cathepsin L could partake in activating these processes has not been previously tested, although the protein has been isolated in the hemocytes (Schleicher, VerBerkmoes, Shah, & Nyholm, 2014).

Because the bacteria‐induced development of the light organ has been extensively studied in this system (McFall‐Ngai, 2014), we have focused here on determining whether Cathepsin L contributes to the cell death underlying this morphogenesis. We first characterized the protein to confirm that its features align with the Cathepsin L of other animals. We then studied the spatiotemporal patterns of transcription of the *cathepsin L* gene, production of the protein, and its activity. The data provide evidence that Cathepsin L is a key player in early development of the *E. scolopes* light organ.

## 2 MATERIALS AND METHODS

### 2.1 General procedures

Our study used the offspring of wild‐caught *E. scolopes* from O'ahu, Hawai'i, USA, which were maintained in the laboratory (Montgomery & McFall‐Ngai, 1993). We compared animals among three treatments: aposymbiotic (“apo”; uncolonized control), symbiotic with wild‐type *V. fischeri* (“sym”), and symbiotic with a *V. fischeri* mutant lacking the lux operon (“Δ*lux*”; Bose, Rosenberg, & Stabb, 2008), with all conditions equally exposed to environmental bacteria. Unless indicated otherwise, we used the standard colonization procedure of approximately 5,000 colony‐forming units per ml of artificial seawater (Ruby & Asato, 1993) and confirmed symbiont infection with a TD 20/20 luminometer (Turner Designs). All animal protocols adhered to the guidelines established by the University of Wisconsin‐Madison. The chemicals were from Sigma‐Aldrich and the molecular reagents and fluorochromes were from Thermo Fisher Scientific unless otherwise specified.

### 2.2 Sequence generation and analysis

We obtained the candidate *cathepsin L* gene transcript (*es‐cathepsin L*) from an RNAseq database, constructed from light organs of juvenile *E. scolopes* at 3 hr postcolonization (Kremer et al., 2013). Additional details, including the handling of tissues, RNA, and cDNA, and obtaining the full‐length cDNA sequence are described in previous studies (Kremer et al., 2013; Peyer, Pankey, Oakley, & McFall‐Ngai, 2014).

With the derived Cathepsin L amino acid sequence (EsCathepsin L), we tested the conservation of functional domains and active sites that are typical of cysteine proteases. We aligned the full sequence in *E. scolopes* with those of representative organisms from the Deuterostomia, Ecdysozoa, and Lophotrochozoa superphyla using CLC Sequence Viewer software (CLC Bio, QIAGEN). With the Simple Modular Architecture Research Tool (SMART; http://smart.embl-heidelberg.de/), we identified the presence and position of the functional domains. Using the Expasy Swiss‐Model (http://swissmodel.expasy.org/), we obtained a predictive model of the mature crystalline structure (Arnold, Bordoli, Kopp, & Schwede, 2006; Biasini et al., 2014; Guex, Peitsch, & Schwede, 2009; Kiefer, Arnold, Künzli, Bordoli, & Schwede, 2009). In addition, we tested for orthology between EsCathepsin L and published sequences as described previously (Peyer, Heath‐Heckman, & McFall‐Ngai, 2017).

### 2.3 In situ hybridization

We used hybridization chain reaction‐fluorescence in situ hybridization (HCR‐FISH) to examine *es‐cathepsin‐L* expression in the light‐organ crypts as a response to symbiosis at 24 hr postcolonization. The protocol enabled us to colocalize the transcript with either wild‐type or Δ*lux V. fischeri* as labeled with 16S ribosomal RNA (Nikolakakis, Lehnert, McFall‐Ngai, & Ruby, 2015). We used a total of five *es‐cathepsin L* probes (Table S1; Molecular Instruments) that had minimal sequence similarity with other host transcripts in the database, all hybridized to the samples for 16 hr at 45°C. To analyze our samples, we used a Zeiss LSM 510 confocal microscope with the gain for all symbiotic light organs normalized to that of aposymbiotic light organs for which the transcript signal was just barely visible. We performed two replicate trials (*n* = 12–20 total animals per condition). All animals for each replicate were from a single clutch so as to minimize variation in expression patterns due to genetic effects.

### 2.4 Immunocytochemistry and western blotting

To examine the presence of EsCathepsin L in host tissues, we performed immunocytochemistry (ICC) using an affinity‐purified polyclonal antibody to Cathepsin L. The antibody was produced in rabbit against the synthetic peptide CVDWRKKGYVTEINKN, located within the protease region, and conjugated to ovalbumin (GenScript). We tested for EsCathepsin L localization in the light organ at 5 and 24 hr postcolonization, and in the mucus that accumulates near the pores. We also tested for the protein in other host tissues. After incubating the samples for 7 days with the primary antibody at a 1:1,000 dilution and the rabbit IgG control at an equivalent concentration, we viewed the tissues by confocal microscopy. To confirm the specificity of the antibody in squid tissues, we performed a western blot analysis using the Cathepsin L primary antibody at 1:5,000 dilution and a rabbit IgG control at an equivalent concentration. We extracted the soluble proteins from the juvenile light organs with phosphate‐buffered saline (50 mmol/L sodium phosphate with 0.1 mol/L sodium chloride, pH 7.4) containing a protease inhibitor cocktail and then used 12.5% SDS‐polyacrylamide gel electrophoresis to separate the proteins. Additional procedures for both ICC and the western blot are included in a previous study and references therein (Kremer et al., 2013).

### 2.5 Enzyme activity assays

We examined EsCathepsin L activity over a range of pH levels to determine if the enzyme was active under conditions typical of the light organ (pH ~5.5 in the crypts and ~6.4 in the mucus; Kremer et al., 2013) and the lysosomes of the cell (usual pH = 3.5–5). Using the same procedure as for western blotting, we extracted the soluble protein from either 25 whole squid or 100 light organs, but into sodium acetate buffer (40 mmol/L, pH 6) instead of PBS and without protease inhibitors. To examine enzyme activity, we modified the methods described previously for Cathepsin L (Barrett & Kirschke, 1981; Chhikara, Mahajan, Gupta, & Chauhan, 2002; Li, Jaffe, Fazleabas, & Verhage, 1991; Mason, Green, & Barrett, 1985). Into a 96‐well plate, we dispensed a total of 53 μl of solution per well that included 25 μg soluble protein, 2 μmol/L DTT, 0.4 mmol/L Rhodamine 110‐based bis‐peptide substrate (CBZ‐Phe‐Arg)_2_‐R11 (Molecular Probes) resuspended in DMSO, and sodium acetate buffer (40 mmol/L), which was suitable over the range of pH levels of 3, 4, 5, 6, and 7. The DTT served as an activation agent and (CBZ‐Phe‐Arg)_2_‐R11 was a fluorescent substrate specific to Cathepsin L. We performed three separate extractions each for whole squid and light organs giving a total of six replicates. For negative controls, we used the same solutions above minus the protein extract and, for a positive control, we replaced the squid protein extract with a human Cathepsin L protein at 1 μg per reaction. With the dispensed solutions loaded onto a plate, we used a Tecan microplate reader at 488 excitation and 525 emission to measure fluorescence over the course of 1 hr until the gain normalized. Activity was measured as fluorescence following cleavage of the (CBZ‐Phe‐Arg)_2_‐R11, which we normalized to that of the negative control containing all solutions except for the protein.

To validate the specificity of the activity detection method, we tested whether the enzyme activity could be quenched with different inhibiting agents. Our inhibitors included: Z‐VAD‐FMK (pan caspase inhibitor at 20, 60, 80 μmol/L; Promega); a Cathepsin L‐specific inhibitor (15, 20, 25 μmol/L; Santa Cruz Biotechnology sc‐361358); and leupeptin (nonspecific cysteine protease inhibitor at 20, 50, 100 μmol/L). In addition, we tested whether coincubation with the *E. scolopes* Cathepsin L antibody would abrogate activity (20 mg ml^−1^; GenScript). Finally, we tested the DMSO (0.25%, 0.5%, 1%), which we used as a solvent for the Cathepsin L‐specific inhibitor and as a tissue permeant in subsequent experiments (see Section 3.4), to ensure that it would have a negligible effect on the enzyme relative to the inhibitors. We followed the same protocol as above with the same solutions, but with the inhibitors included and using proteins from light organs only. Our negative controls consisted of all solutions except for the protein. For comparison with the *E. scolopes* Cathepsin L antibody as an inhibitor, we used the IgG control. For each inhibiting agent, we performed three separate protein extractions for three replicate tests. In all samples, we used sodium acetate buffer (40 mmol/L) at a pH of 6, which was within the pH range described for the light organ environment (pH 5.5–6.4; Kremer et al., 2013). We calculated relative percentage fluorescence by normalization of the inhibited solutions to those containing no inhibitors.

In addition to examining EsCathepsin L activity in extracted proteins, we tested for activity in live squid tissues during the colonization process. We preincubated three to four juveniles for 1 hr in 2 ml artificial seawater with 2 μmol/L (CBZ‐Phe‐Arg)_2_‐R11 and 1% DMSO, and then colonized with 10^5^ colony‐forming units of RFP‐labeled *V. fischeri* per ml of artificial seawater. After ~1 hr of colonization, we used 20 μg ml^−1^ wheat germ agglutinin Alexa 633 (WGA; Vector Laboratories) for 5 min to stain the host mucus in which bacterial aggregates form. We also examined squid left uncolonized, but exposed to environmental bacteria naturally occurring in the artificial seawater. We anesthetized the squid in 2% ethanol for viewing EsCathepsin L activity during the colonization process by confocal microscopy. In a separate experiment, we replaced the (CBZ‐Phe‐Arg)_2_‐R11 with 50 nmol/L LysoTracker Green DND‐26 for 30 min to stain the acidic organelles, enabling us to determine if bacterial aggregates associate with acidifying cells in the mucus and superficial host tissues. Finally, we examined EsCathepsin L activity after ~5 and 24 hr of colonization using the standard 5,000 colony‐forming units per ml of artificial seawater. In this experiment we incubated the juveniles in 3 ml artificial seawater, 2 μmol/L (CBZ‐Phe‐Arg)_2_‐R11, and 1% DMSO and examined various tissues in the squid in addition to the light organ. At 5 hr we used 50 nmol/L LysoTracker Red DND‐99 to stain the acidic organelles and at 24 hr we used WGA to stain the mucus, 30‐ and 5‐min incubation times, respectively.

### 2.6 Inhibition of EsCathepsin L and developmental cell death

We also probed for a functional role of EsCathepsin L during the cell death program that ensues once the symbiosis is established. We tested whether inactivation of EsCathepsin L could affect cell death in the host using the same pharmacological inhibitors described above: Z‐VAD‐FMK (20, 60, 80 μmol/L); a Cathepsin L‐specific inhibitor (15, 20, 25 μmol/L); and leupeptin (20, 50, 100 μmol/L). Each treatment of ~10 squid consisted of 10 ml artificial seawater, 1% DMSO as a tissue permeant, and an inhibitor of the given concentration. We preincubated the squid for ~2 hr and then followed with 3 hr of colonization with *V. fischeri*. After refreshing the solutions to remove excess bacteria, we continued the incubation with the antibody for an additional 20 hr. For controls, we used squid both colonized in artificial seawater under normal laboratory conditions to confirm the use of healthy animals and those with the added 1% DMSO. In testing all conditions for each inhibitor, we collected squid from a single clutch, performing three replicate experiments. As a complementary experiment, we tested whether the *E. scolopes* Cathepsin L antibody (GenScript) could render the protein inactive through steric hindrance, thereby serving as an alternative inhibitor that was unlikely to induce side effects that can occur with pharmacological agents. This experimental setup was identical to that with the inhibitors above, except that we used only one concentration for the antibody (20 mg ml^−1^) and we used rabbit IgG at an equivalent concentration as an additional control.

Within the anterior appendages of the light organ undergoing symbiont‐induced regression (Figure 1b and c), we examined the effect of EsCathepsin L inhibition on developmental cell death. We anesthetized the colonized juveniles in 2% ethanol and preserved them in 4% paraformaldehyde and mPBS (50 mmol/L sodium phosphate, pH 7.4, 0.4 mol/L NaCl) for 18 hr at 4°C with gentle mixing. We washed the samples (4 × 30 min) in mPBS and followed with tissue permeabolization in 1X mPBS with 1% Triton‐X100 for 2 days at 4°C with gentle mixing and subsequent washes in 1X mPBS (2 × 30 min). We performed the TUNEL assay on light organs per manufacturer specification (Promega), counterstained the nuclei with TOTO‐3, and scored late‐stage cell death by counting TUNEL+ nuclei of one whole anterior appendage (Figure 1b and c) using a confocal microscope. In an additional experiment, we tested whether EsCathepsin L participates in cell death through a pathway dependent or independent of symbiont luminescence. Thus, we examined juveniles colonized with Δ*lux V. fischeri*, following the same protocol as above, but using only the antibody to deactivate the EsCathepsin L protein.

Using the statistical package R (Core Development Team, 2008), we tested whether cell death differed among conditions. We used an analysis of variance (ANOVA) to test whether the number of TUNEL+ nuclei depended on colonization condition and replicate. The model for cell death, *D*, as the dependent variable was as follows:

$$D=\beta_{0}+\beta_{1}x_{c}+\beta_{2}x_{r},$$

where independent variables were colonization condition (*x*_*c*_) and replicate (*x*_*r*_). The variable *x*_*r*_ represented the three independent biological replicates. Maximum likelihood parameter estimates were β_0_, β_1_, and β_2_. To obtain normal distributions, we used Box‐Cox transformations in R using maximum likelihood to determine the optimal power transformations for *D*. To obtain the model that best fit the data, we used a model‐selection approach with backward selection and an *F*‐test to evaluate the significance of removing replicate as a factor (Crawley, 2008). We performed Tukey post hoc pairwise comparisons for *D* between the different conditions and treated all variables as fixed effects.

## 3 RESULTS

### 3.1 Characterization of the EsCathepsin L sequence

Analysis of the derived EsCathepsin L sequence revealed regions that characterize the protein: a signal peptide at the N‐terminus, a propeptide inhibitor domain, and a papain‐family cysteine protease domain (Figure S1a). The putative cleavage site at the beginning of the protease region aligned with that of other organisms. The protease region further contained three highly conserved thiol subregions, cysteine, histidine, and asparagine, with predicted active residues (Arockiaraj et al., 2013; Ma et al., 2010). Several Cathepsin L motifs ERFNIN, GNFD, and GCNGG (Ma et al., 2010; Niu et al., 2013) were also present in EsCathepsin L, although the first two were not fully conserved. Overall, the protein showed a relatively high degree of conservation in its tertiary structure (Figure S1b).

In a phylogenetic reconstruction, EsCathepsin L clustered with other members of the Lophotrochozoa superphylum and was most closely related to *Octopus bimaculoides*, another cephalopod mollusk (Figure S1c). The members of the Ecdysozoan clade clustered together while those of the Deuterostomia formed different groupings.

### 3.2 Localization of the transcript and protein in response to symbiosis

**Figure 2:**
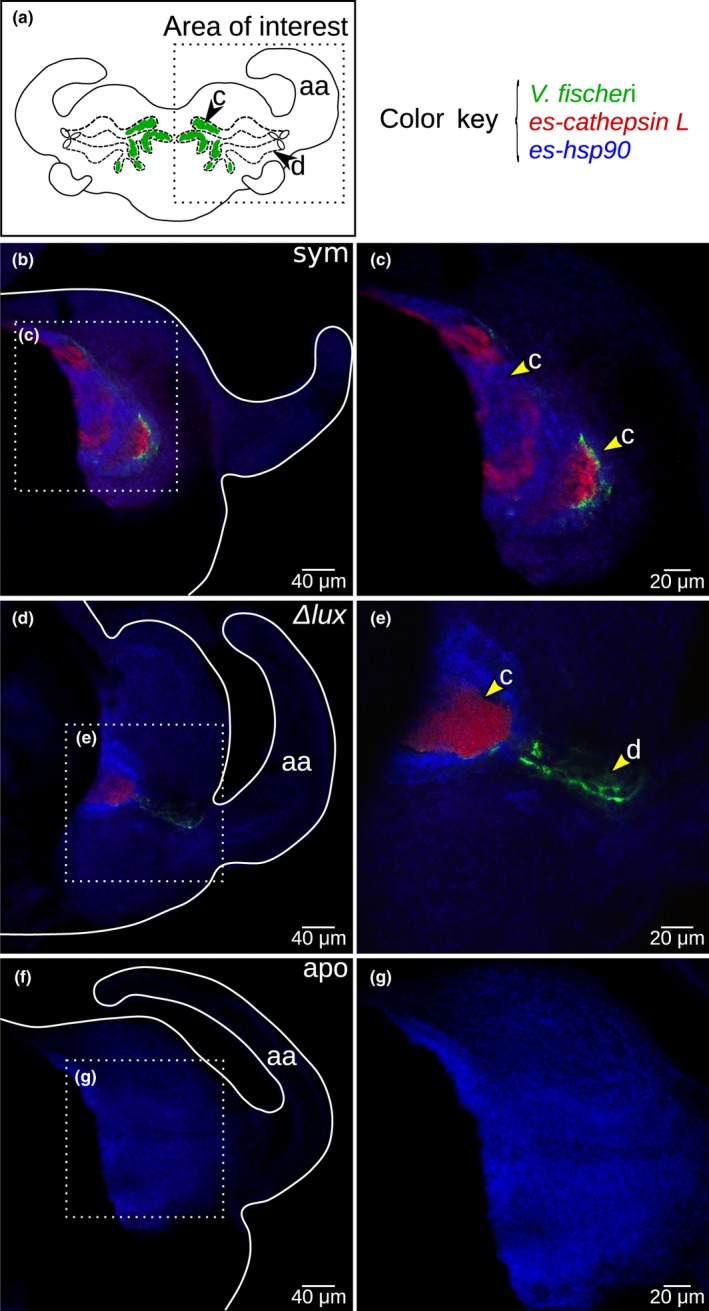
Expression of *es‐cathepsin L* by hybridization chain reaction‐fluorescence in situ hybridization at 24 hr postcolonization. (a) The region of transcript expression in the light organ was within the crypts (c) where *V. fischeri* resides. The anterior appendage (aa) and duct (d) are included for reference. (b–e) *V. fischeri* labeling is shown with *es‐cathepsin L* in the crypts of light organs symbiotic with wild‐type (b, c; “sym”) and Δ*lux V. fischeri* (d, e; “Δ*lux*”). Some Δ*lux V. fischeri* are visible in both the crypt and duct. (f, g) Light organs aposymbiotic with no *V. fischeri* (“apo”) show no transcript expression as compared to symbiotic light organs at an equivalent gain. *E. scolopes* heat‐shock protein 90 transcript (“*es‐hsp90*”), which occurs in all cells, was used only as a counterstain.

Examination of the *es‐cathepsin L* transcript in the light organ showed labeling in response to symbiosis. Within the crypts (Figure 2a), the *es‐cathepsin L* transcript co‐occurred with the 16S ribosomal RNA signal from *V. fischeri* cells, as demonstrated with HCR‐FISH methods (Figure 2b and e). Specifically, expression of *es‐cathepsin L* was visible in crypts harboring both wild‐type (Figure 2b and c) and Δ*lux V. fischeri* (Figure 2d‐e) as compared to light organs of aposymbiotic animals (Figure 2f and g).

**Figure 3:**
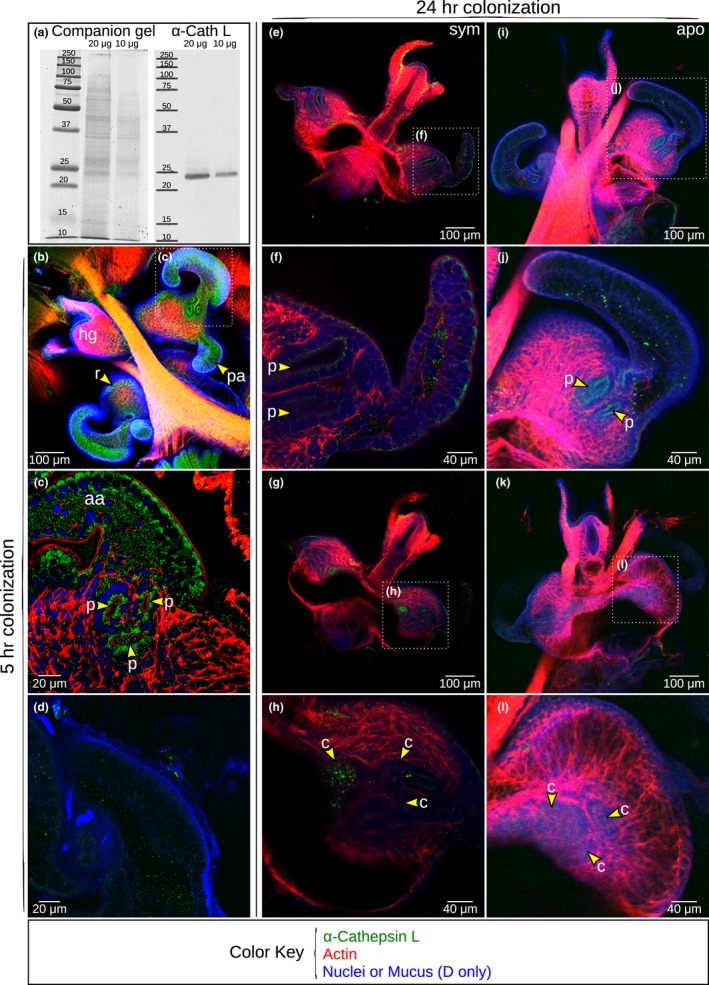
Western blotting and localization of EsCathepsin L in the light organ by immunocytochemistry. (a) The western blot indicated cross reactivity of the *E. scolopes* Cathepsin L antibody with a protein in the light organ at the predicted molecular mass of ~23.5 kDa without the propeptide. The soluble protein fraction of 10 and 20 μg is shown on the gel (left, companion gel) and transfer membrane with the Cathepsin L antibody (right). Standards are indicated in kDa. (b, c) At 5 hr postcolonization, the protein localized by immunocytochemistry to the superficial epithelial cells of the tissues where specificity is determined and where cell death occurs, including the anterior appendage (aa), posterior appendage (pa), ciliated ridge (r), and pores (p). Hindgut (hg) included for reference. (d) The protein was also present in the mucus produced on the surface of the light organ. (e, f) At 24 hr postcolonization, the protein appeared less visible on the surface, but was present in the sinuses and (g, h) crypts (c) of symbiotic (“sym”) light organs. (i‐l) Although still present in surface tissues of aposymbiotic (“apo”) light organs (i, j), the protein remained absent in the crypts (k, l). Counterstains: rhodamine phalloidin for actin, TOTO‐3 for nuclei, and WGA for the mucus

In addition to the transcript, we tested for the presence of the protein in the light organ. Initially, we tested for cross reactivity between our purified Cathepsin L antibody and the protein in *E. scolopes*. A western blot indicated cross reactivity of the antibody with a protein in the soluble fraction at ~23.5 kDa, the predicted molecular mass of EsCathepsin L without the propeptide (Figure 3a). Occasionally the western blots have an additional band at ~36.2 kDa, which is likely the inactive, propeptide form of the protein with the inhibitory domain, but without the signal peptide. The signal peptide is cleaved upon secretion of the protein. Localization of the protein by ICC indicated labeling around the pores and within the appendages and ciliated field at 5 hr postcolonization (Figure 3b and c). The protein was present in the cytosol of the cell. Labeling was also present in the mucus that is produced by the host upon exposure to MAMPs in the seawater (Figure 3d). At this earlier phase of colonization, patterns of protein localization were indistinguishable between aposymbiotic and symbiotic light organs. By 24 hr postcolonization the protein was diminished, although still present, in all superficial tissues of symbiotic light organs (Figure 3e and f) and became detectable in the crypt spaces (Figure 3g and h). Aposymbiotic light organs retained some protein message in the superficial tissues (Figure 3i and j), but showed no signal in the crypts at 24 hr (Figure 3k and l). All IgG controls were free of labeling (Figure S2).

In a comparative experiment, we examined protein localization to other tissues in the squid body (Figure S3a). Signal was present throughout the gills and was striking within the nearby ventricle, or heart (Figure S3b and c). Faint labeling also occurred in the head between the eyes and optic lobes (Figure S3d), and throughout the digestive gland (Figure S3e). Finally, the tentacles (Figure S3f) and mantle (Figure S3g) exhibited a moderate degree of labeling. In all these tissues the IgG controls showed undetectable labeling (Figure S4).

### 3.3 Activity of EsCathepsin L in vivo and in vitro

**Figure 4:**
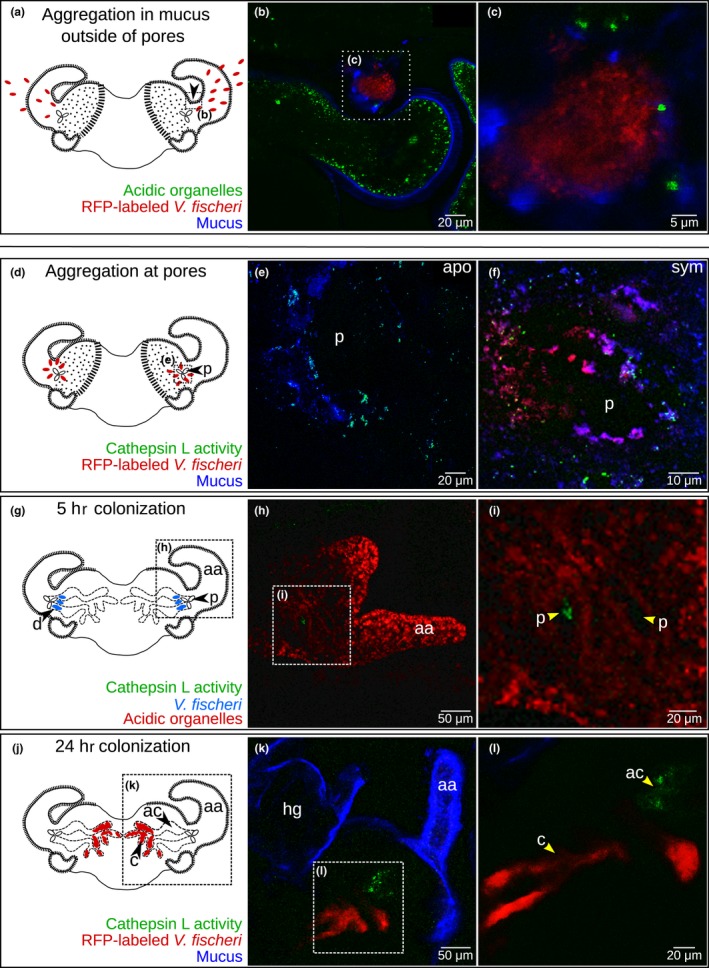
Early colonization of the light organ and EsCathepsin L activity. The sites of interest are indicated with a black arrow (see colonization schemes a, d, g, and j). (a) During the first stage of colonization, *V. fischeri* (red ovals) encounters the ciliated field on the surface of the light organ. (b, c) The symbionts aggregated within the mucus secreted by the host (b), which was associated with acidic organelles (c). (d) After initial aggregation, the symbionts (red ovals) collect in the cilia surrounding the pores (p). (e, f) Around the pores, EsCathepsin L was active in the cilia or associated mucus whether exposed to environmental bacteria alone (e; “apo”) or *V. fischeri* (f; “sym”). (g) After ~5 hr of colonization *V. fischeri* (blue ovals) has bypassed the cilia‐encircled pores and proceeds into the ducts (d). For reference, the *V. fischeri* cells are only indicated in scheme g (i.e., not in H or I). (h, i) EsCathepsin L was active within the ducts just inside the pores. Anterior appendage (aa) included for reference. (j) Finally within ~24 hr of colonization, *V. fischeri* (red ovals) gains entry into the crypts (c). (k, l) EsCathepsin L was active in the antechamber (ac) adjacent to the crypts and resident bacteria. Anterior appendage (aa) and hindgut (hg) included for reference. Counterstains: LysoTracker Green or LysoTracker Red for the acidic organelles and WGA for the mucus

We were interested in knowing whether EsCathepsin L was active in vivo during *V. fischeri* colonization. Initially, we examined whether the host tissues offer a suitable environment for activation of the protein. *V. fischeri* from the seawater first aggregates outside of the pores within the mucus that is secreted by the host (Figure 4a). Here our images revealed patches of low pH within the mucus (Figure 4b and c), and thus, where EsCathepsin L could be active.

Our biochemical data confirmed that during the early phases of *V. fischeri* colonization, EsCathepsin L was active in vivo throughout the light organ (Figure 4d‐l). Surrounding the pores (Figure 4d), an enzyme assay detected EsCathepsin L activity in aposymbiotic (Figure 4e) and symbiotic light organs (Figure 4f), both treatments containing nonspecific environmental bacteria. At ~5 hr postcolonization with *V. fischeri* (Figure 4g), activity of EsCathepsin L was visible just inside the pores (Figure 4h and i). By 24 hr postcolonization (Figure 4j), EsCathepsin L was active in the antechamber, adjacent to the bacteria‐filled crypts (Figure 4k and l). We did not observe EsCathepsin L activity in the appendages where cell death occurs, therefore the protein might have been present in the inactive propeptide form. These tissues were abundant with acidic organelles (Figure 4h and i) and labeling of the protein with the antibody was coincident with these regions of the cells (Figure S5), suggesting the potential for enzyme activation. Of the additional tissues of the squid body that produced EsCathepsin L (Figure S3), we observed activity within the gills and digestive gland (Figure S6). In general, activity of EsCathepsin L in the light organ and other tissues either coincided with the protein by ICC (Figures 3, S3) or acidic organelles where the protein could be activated (Figure 4, S5).

**Figure 5:**
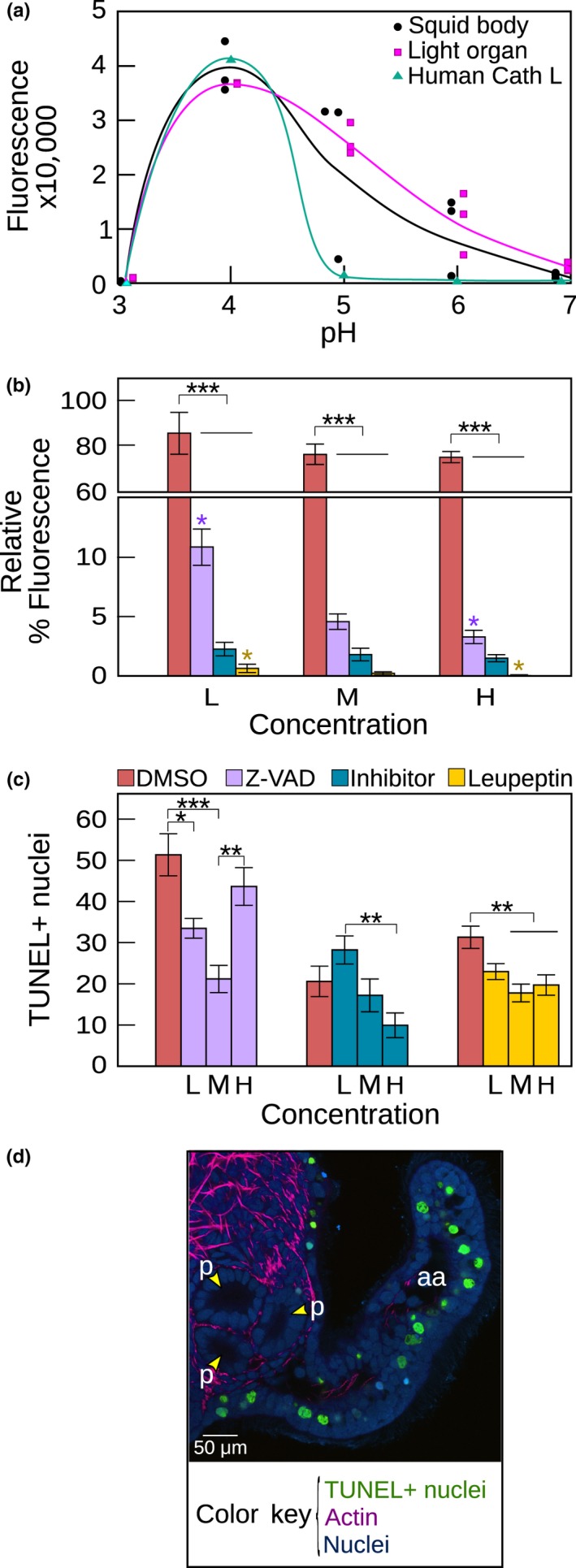
Activity and inhibition of EsCathepsin L in squid tissues. (a) Enzyme activity (as fluorescence) over a range of pH values. The human Cathepsin L was used as a positive control. Data consist of three replicates of the whole squid body, three replicates of the light organ, and one replicate of the human Cathepsin L protein at each pH level. The regression curves serve as visual guides only. (b) Enzyme activity (as % fluorescence relative to the control that excludes inhibitors) in the presence of a caspase (Z‐VAD‐FMK; 20, 60, 80 μmol/L), Cathepsin L‐specific (15, 20, 25 μmol/L), and cysteine protease inhibitor (leupeptin; 20, 50, 100 μmol/L) at increasing concentrations (L: low, M: medium, H: high). Dimethyl sulfoxide (DMSO, 1%) was tested because it served as a solvent for the Cathepsin L‐specific inhibitor and a tissue permeant in experiments involving cell death (see Figure 6). Purple and yellow * indicate significant differences in activity between the low and high dosages for the Z‐VAD‐FMK and leupeptin treatments, respectively. (c) Number of dying cells (as TUNEL+ nuclei) in the presence of inhibitors at increasing concentrations (see also b). Each inhibitor was tested with a different cohort of squid, on different days, and are thus independent of one another. Error bars are standard error of the mean. (***P < .001; **.001 < *P* < .01; *.01 < *P* < .05). (d) Light organ anterior appendage (aa) showing TUNEL+ nuclei. Counterstains: rhodamine phalloidin for actin and TOTO‐3 for nuclei

We confirmed that EsCathepsin L was active under acidic conditions by in vitro examination of the enzyme over a range of pH levels. Using extracted protein samples from light organ and whole body tissues, EsCathepsin L exhibited the highest activity over a pH range of 4–6 (Figure 5a). Peak activity occurred at a pH of ~4, the same as our positive control, human Cathepsin L, that had a more narrow range of activity.

EsCathepsin L activity in vitro was significantly suppressed by a caspase (Z‐VAD‐FMK), Cathepsin L‐specific, and cysteine protease (leupeptin) inhibitor (ANOVA: *F *=* *132.3, df = 11, *P* < 2e‐16; Figure 5b). For each inhibitor at every dosage, enzymatic activity was significantly lower than the DMSO (dimethyl sulfoxide) control (Tukey: *P* < .00001, DMSO versus each inhibitor; Figure 5b). The Z‐VAD‐FMK and leupeptin inhibitors showed dose‐dependent responses with activity associated with the high dose significantly lower than that of the low dose (Tukey: Z‐VAD‐FMK, *P = *.021; leupeptin, *P = *.018), whereas the DMSO and Cathepsin L‐specific inhibitor exhibited dose‐independent responses (Tukey: DMSO, *P = *1.0 all dose comparisons; Cathepsin L‐specific inhibitor, *P = *1.0 all dose comparisons; Figure 5b). The antibody to EsCathepsin L failed to inhibit the enzyme in this assay, as activity with the antibody relative to the IgG control was 110%.

### 3.4 Effects of EsCathepsin L inhibition on developmental cell death

Following inhibition of EsCathepsin L, we found evidence for involvement of the protein in developmental cell death. In a first set of experiments, we observed a treatment effect for all three pharmacological inhibitors (ANOVA: Z‐VAD‐FMK *F *=* *8.9, df = 3, *P = *7.1e‐05; Cathepsin L‐specific inhibitor *F *=* *5.3, df = 3, *P *=* *.0031; leupeptin *F *=* *6.8, df = 3, *P *=* *.00039; Figure 5c and d). Relative to the DMSO control, the number of TUNEL+ nuclei was significantly lower in the presence of Z‐VAD‐FMK for the low and medium concentrations (Tukey: low *P *=* *.021; medium *P *=* *.000047) and leupeptin for the medium and high concentrations (Tukey: medium *P *=* *.0032; high *P *=* *.0028) (Figure 5c and d). For the Cathepsin L‐specific inhibitor the number of TUNEL+ nuclei was significantly lower for the high relative to the low concentration (Tukey: *P *=* *.0016). We also observed a replicate effect for all three pharmacological inhibitors (ANOVA: Z‐VAD‐FMK *F *=* *24.6, df = 3, *P *=* *4.1e‐10; Cathepsin L‐specific inhibitor *F *=* *9.5, df = 3, *P *=* *4.5e‐05; leupeptin *F *=* *4.2, df = 3, *P *=* *.008), suggesting that not all clutches responded in consistent ways for all concentrations. Thus, while the pharmacological agents had an inhibiting effect on cell death, they might influence more than one pathway or have a slight toxicity that can affect the cells in unpredictable ways that extend beyond the normal developmental program in our study system.

**Figure 6:**
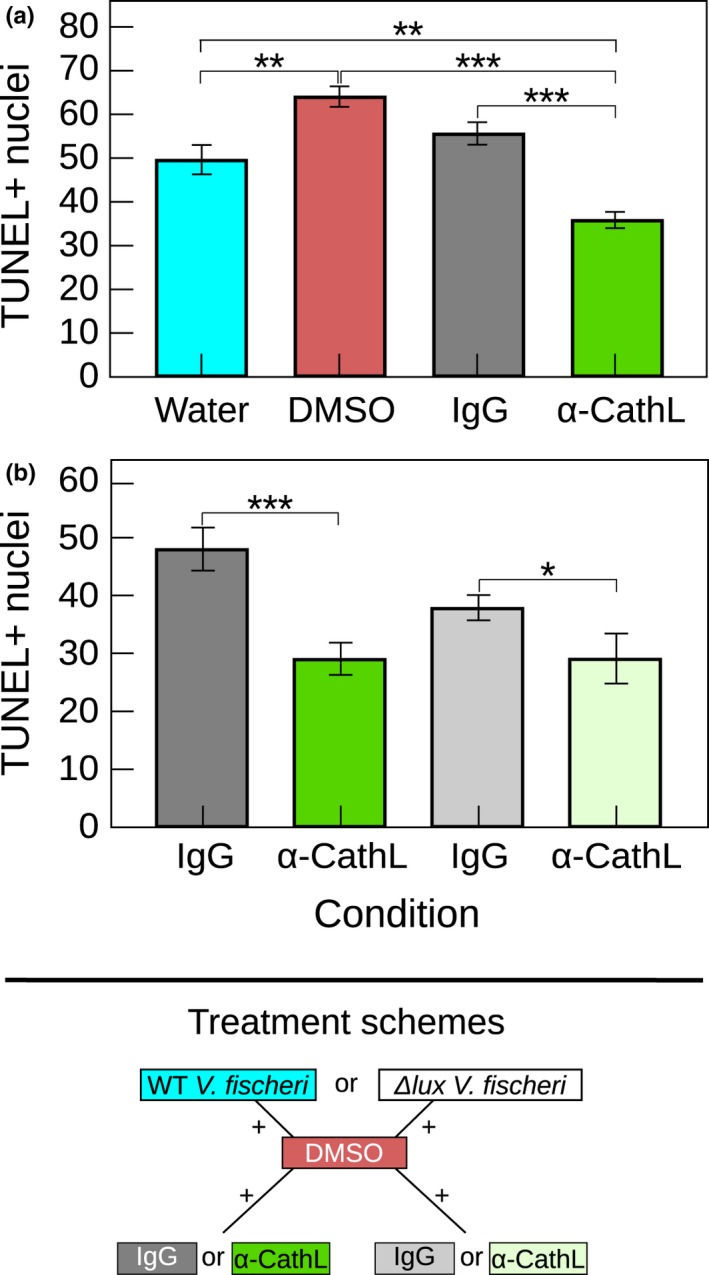
Evidence for EsCathepsin L involvement in cell death, acting in part through a light‐independent pathway. (a) Number of dying cells (as TUNEL+ nuclei) in animals after 3 hr incubation with wild‐type *V. fischeri* and 24 hr exposure to the primary treatment, the *E. scolopes* Cathepsin L antibody (α‐CathL), compared with three controls. The IgG control was the most comparable to the antibody treatment. (b) TUNEL+ nuclei in animals colonized with either Δ*lux* or wild‐type *V. fischeri* (as an experimental control) for 24 hr while incubating in the *E. scolopes* Cathepsin L antibody or IgG control. In both graphs, the DMSO (1%) permeabolized the tissues to assist antibody penetration and error bars are standard error of the mean. (***P < .001; *.01 < *P* < .05)

To bypass the potential negative effects of the pharmacological inhibitors, we ran a second set of experiments, testing the inhibitory effects the *E. scolopes* Cathepsin L antibody on cell death (Figure 6). Exposure to the antibody significantly decreased cell death (ANOVA: *F *=* *24.3, df = 3, *P* < .00001; Figure 6a). Specifically, animals exposed to the antibody had significantly fewer TUNEL+ nuclei than those exposed to all control treatments, including IgG (Tukey: *P* < .00001), DMSO (*P* < .00001), and artificial seawater alone with *V. fischeri* (*P *=* *.0012).

We further determined that the mechanism by which EsCathepsin L is involved in cell death is independent of symbiont luminescence, at least to some degree. Similar to animals colonized with wild‐type *V. fischeri*, those harboring Δ*lux V. fischeri* exhibited a significant decrease in cell death when exposed to the antibody (ANOVA: *F *=* *9.5, df = 3, *P = *.000013; Figure 6b). For these animals, the number of TUNEL+ nuclei was significantly lower in the antibody relative to the IgG control treatment (Tukey: *P* < .05).

## 4 DISCUSSION

We examined EsCathepsin L in the *E. scolopes*–*V. fischeri* symbiosis, focusing on the involvement of the protein in host developmental cell death. We found support for EsCathepsin L as a participant in the loss of cells comprising the superficial light‐organ tissues, which are employed only at the onset of symbiosis. Our conclusion is based on in silico, in vitro, and in vivo experiments that confirm protease activity at pH levels relevant to the system and attenuation of cell death following protein inhibition.

### 4.1 Sequence characteristics, activity, and behavior of EsCathepsin L

In silico analysis of the EsCathepsin L sequence confirmed the presence of features that characterize this group of cysteine proteases (Figure S1). The signal peptide indicated that EsCathepsin L is likely to be secreted out of a cell (Figure S1a), with the protein in both the mucus (Figure 3d) and crypt spaces (Figure 3g and h) lending support for such secretion. The propeptide inhibitor domain suggests that EsCathepsin L remains inactive until cleaved at the conserved putative site where the protease region begins (Figure S1a; Ma et al., 2010; Niu et al., 2013). The signature for protease activity includes four key cysteine residues and an oxyanion hole, which assists in catalyzing enzymatic reactions (Figure S1a; Ma et al., 2010; Arockiaraj et al., 2013). The grouping of EsCathepsin L with other molluscan Cathepsin L proteins in our phylogenetic reconstruction offered another level of support for conserved function among the lophotrochozoans (Figure S1c).

Further investigation of EsCathepsin L in vitro demonstrated that the protease could be activated in a low pH environment and quenched with select inhibitors. Peak activity occurred within the range of pH levels typical of lysosomes (~3.5–5; Figure 5a). Although this peak was lower than the previously measured pH levels of the light organ mucus (~6.4) and crypts (~5.5) (Kremer et al., 2013), the enzyme retained activity at these slightly higher levels (Figure 5a). The activity with varying pH levels is remarkably similar to that of Cathepsin L in vertebrate tissues, including human liver (Mason et al., 1985) and cat uterine flushings (Li et al., 1991). Enzyme activity could be suppressed most dramatically by a cysteine protease inhibitor (leupeptin), but also one specific to Cathepsin L and another as a general caspase inhibitor (Z‐VAD‐FMK) (Figure 5b). Our combined findings that protein activity occurred at low pH and could be diminished by select inhibitors offers evidence that EsCathepsin L as a cysteine protease has predictable behavior. Adding further support, we confirmed EsCathepsin L activity in vivo by microscopy examination during early colonization of the light organ by *V. fischeri* (Figure 4). The low pH environment surrounding the superficial light‐organ tissues (Figure 4a–c) indicated that the protein could be activated, which we observed in the mucus (Figure 4d–f) and within and around the pores (Figure 4g–i).

### 4.2 Function of EsCathepsin L in developmental cell death

**Figure 7:**
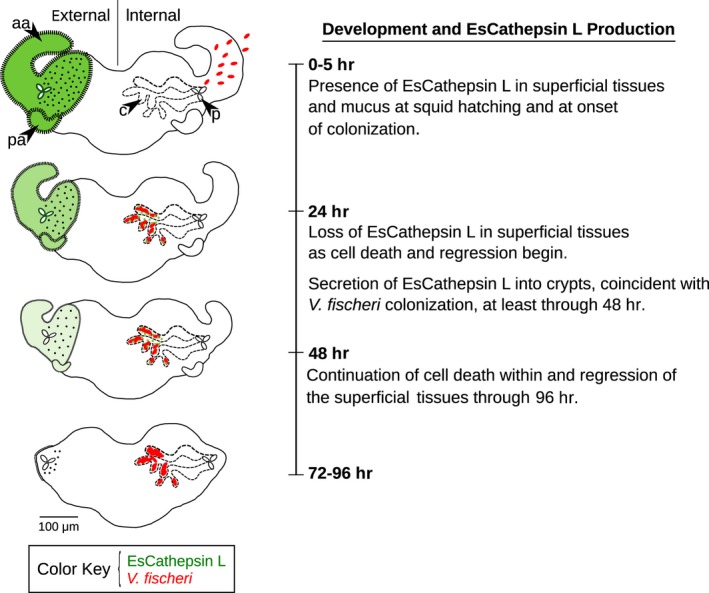
Summary of EsCathepsin L and developmental dynamics in the symbiotic light organ. Within the first few hours of hatching, EsCathepsin L (green) localized to the superfical tissues of the light organ, including the anterior and posterior appendages (“aa,” “pa”), pores (“p”), and externally secreted mucus. By 24 hr the protein began to decrease in the superficial tissues, corresponding with the onset of cell death within and regression of the appendages, which continues through ~96 hr. Secretion of the protein into the crypts (“c”) occurred, coinciding with *V. fischeri* colonization (red ovals)

Our data provided a case for the involvement of EsCathepsin L in developmental cell death of the light organ following colonization with *V. fischeri*. Supporting results first included positioning of the protein in tissues involved in development, namely the anterior and posterior appendages (Figures 3b–f, 7). The protein, while in abundance several hours after hatching (Figures 3b and c, 7), was diminished within these sites in symbiotic light organs by 24 hr (Figures 3e and f, 7), a time point by which cell death is fully underway and the appendages are poised for regression. At this same time point EsCathepsin L was also visible in the appendages of aposymbiotic animals (Figure 3i and j). The protein might have been in its inactive form, because we did not detect activity within these tissues. However, the protein colocalized with acidic organelles in these tissues (Figure S5), suggesting the potential for such activation, and acidification is a key indicator of cells undergoing death in this system (Foster & McFall‐Ngai, 1998). EsCathepsin L was also present within (Figure 3g and h) and active in the vicinity of the colonized crypts (Figures 4j–l, 7). The role of the protein in the crypts is unclear; it might be part of a signaling cascade, as the message for cell death in the superficial tissues is sent once the symbiont has colonized the crypts (Doino & McFall‐Ngai, 1995), or serve in immune function, but additional research would be required to test either of these hypotheses.

Inhibition of EsCathepsin L by both the pharmacological agents and the antibody to EsCathepsin L, and subsequent attenuation of cell death up to ~50% further linked the protease to the cell death process (Figure 6a; see Repnik et al., 2012). If EsCathepsin L were solely involved in processing the degenerating tissues (e.g., through hemocytes; Schleicher et al., 2014), the inhibitors should not have influenced the number of dying cells. Further support comes from recent work showing that matrix metalloproteinases in *E. scolopes* are the principle elements degrading the extracellular matrix, allowing cells to slough from the surface (Koropatnick et al., 2014). We were unable to fully halt cell death, implying that the inhibitors did not completely penetrate the superficial tissues and block key sites or that more than one pathway contributes to cell death in this system, possibly involving multiple caspases or Cathepsin proteins. For instance, Cathepsin L is linked to apoptosis in midgut development of the bollworm *Helicoverpa armigera* by increasing the activity of caspase‐1 (Yang et al., 2017), whereas in the tussar moth *Antheraea pernyi*, knock down of Cathepsin L reduces *caspase‐3* transcript expression, which is also thought to affect metamorphosis (Sun et al., 2018). Like Cathepsin L, the cysteine protease Cathepsin B is also involved in apoptosis (Fonović & Turk, 2014). In murine models focused on the digestive system, where cysteine proteases are produced in abundance, Cathepsins L is an antagonist to Cathepsin B, the deletion of which inactivates caspase‐3 and apoptosis‐inducing factor (Sendler et al., 2016). If Cathepsins L and B are similarly antagonistic in *E. scolopes*, such interactions might have limited the extent to which the nonspecific cysteine protease inhibitor leupeptin could decrease cell death in the light organ. Of all the inhibitors that we used, the antibody to EsCathepsin L was the least likely to induce confounding pharmacological phenotypes. The most plausible mechanism of inhibition was steric hindrance in the cell, where the antibody directly interacts with relatively large substrates such as proteins. The lack of antibody inhibition of Cathepsin L activity in tissue extracts (i.e., no change in cleavage of the fluorescent substrate [(CBZ‐Phe‐Arg)_2_‐R11]; see Section 4.2) was not necessarily surprising. It is not uncommon for antibodies to perform differently under different circumstances (ICC vs. Western *vs*. activity assays). In this case, the antibodies did not function the same with extracted, total soluble protein, as they did within the tissues. The tissue environment is certainly different; for example, the protein–protein interactions of the tissues will be disrupted in extracts, and the cell is a more reducing environment, where antibodies may have more access to the active sites that would disrupt ligand binding by the enzyme.

The light organ shares a number of features with the eye (Crookes et al., 2004; Montgomery & McFall‐Ngai, 1992; Peyer et al., 2014, 2017; Tong et al., 2009), as both sets of tissues are receivers of light, albeit from different sources. While studies are few, inhibition of Cathepsin L also preserves other light‐sensitive cells including the photoreceptors in *norpA* mutant *Drosophila melanogaster* that experience light‐dependent retinal degeneration (Kinser & Dolph, 2012). In the squid host, cell death has been found to occur in response to symbiont luminescence in addition to MAMPs (Koropatnick et al., 2007; McFall‐Ngai, Heath‐Heckman, Gillette, Peyer, & Harvie, 2012). Our data suggest that EsCathepsin L acts, at least in part, through a light‐independent pathway, as inhibition of the protease partially blocked cell death in animals with *Δlux V. fischeri* that produce no light (Figure 6b). Thus, any cell death signaling that might involve EsCathepsin L has the potential to occur with or without symbiont luminescence. Past studies of the system also demonstrate the occurrence of cell death in the presence of Δ*lux V. fischeri*, although to a lesser degree than with the wild‐type, luminous strain (McFall‐Ngai et al., 2012).

### 4.3 Additional roles for EsCathepsin L

In addition to facilitating developmental cell death, EsCathepsin L is likely to serve other functions in the *E. scolopes*–*V. fischeri* symbiosis. The protein was present in other tissues that associate with bacteria (e.g., gills, digestive gland, tentacles, mantle; Figure S3b–g), with confirmed activity in the gills and digestive gland (Figure S6b and c). None of these tissues experience developmental cell death analogous to the light organ following symbiont colonization; thus, examination of these tissues might offer insights into the role of EsCathepsin L in other processes, possibly pertaining to resident bacteria. In other systems, Cathepsin L is active within equally diverse tissues, sometimes exhibiting antimicrobial properties or activating proteins that modulate the bacterial residents (Byeon et al., 2015; Schleicher et al., 2014; Wang & Sun, 2015). In *E. scolopes*, the protein was active in the light organ along the colonization path and was especially visible in the antechamber where the microbicide nitric oxide persists (Davidson, Koropatnick, Kossmehl, Sycuro, & McFall‐Ngai, 2004) and EsCathepsin L might play a participatory role in the antimicrobial process. Alternatively, EsCathepsin L might assist in the degradation of proteins, such as trypsin and its precusor trypsinogen, as is known to occur in murine models (Sendler et al., 2016). In response to symbiosis, the transcripts are simultaneously upregulated for EsCathepsin L and EsChymotrypsin (Kremer et al., 2013), a serine protease that assists in the hydrolysis of proteins and is activated by trypsin. Similarly, EsCathepsin L might assist in digesting proteins of nutritional benefit to the host or the microbes themselves, providing a key mechanism for energy acquisition in a juvenile with limited time to secure its first meal (see also Hawkins & Day, 1996). In the *E. scolopes*–*V. fischeri* system, a comparative study of all tissues producing EsCathepsin L might yield an insightful function of the protein in the survival and longevity of the host.

## ACCESSION NUMBER

The GenBank accession number of EsCathepsin L is MF765754.

## CONFLICT OF INTEREST

The authors declare no conflict of interest.
