# Supplementary figures and images for: Involvement of a host Cathepsin L in symbiont‐induced cell death
Source: Microbiologyopen. 2018 Apr 24;7(5):e00632. doi: 10.1002/mbo3.632 (PMC6182562; doi:10.1002/mbo3.632)

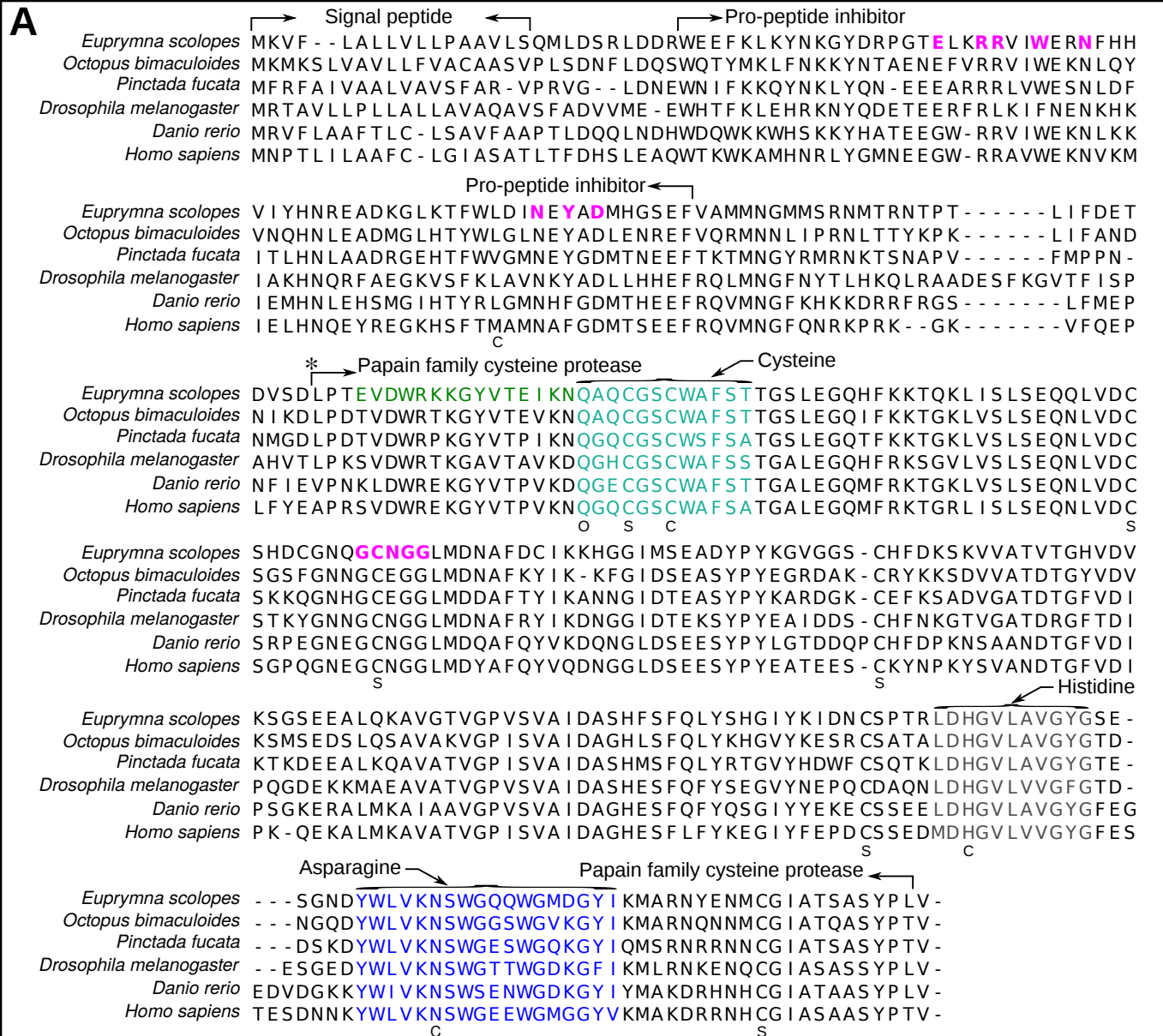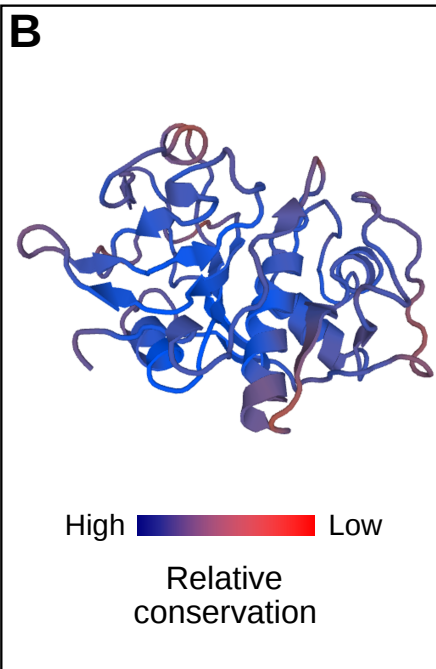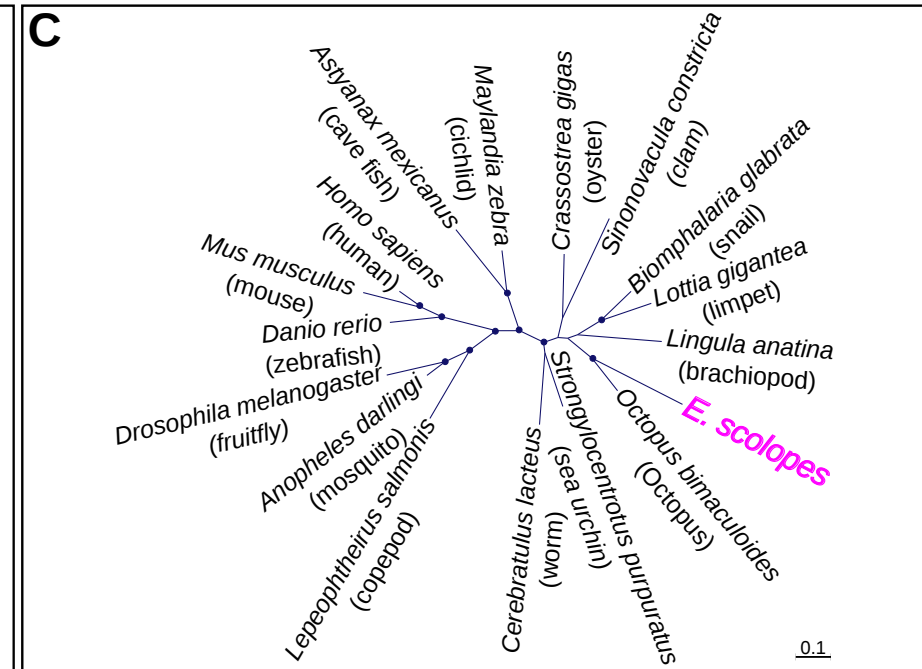

Supplement: Supplementary file 1 [file MBO3-7-e00632-s001.pdf]

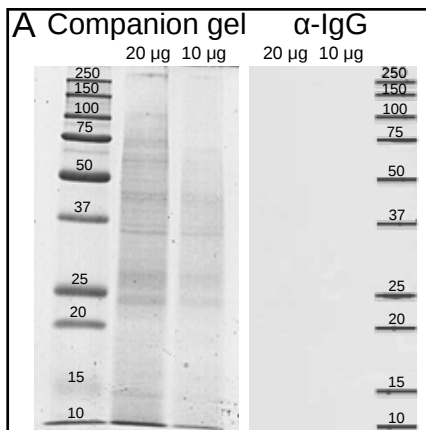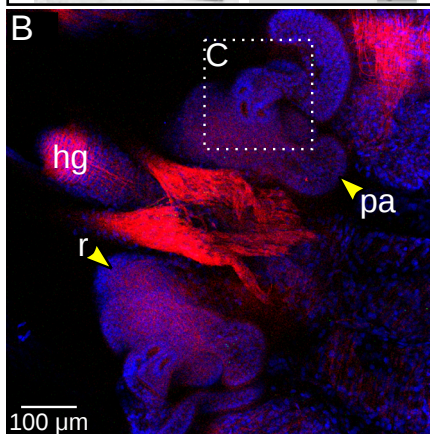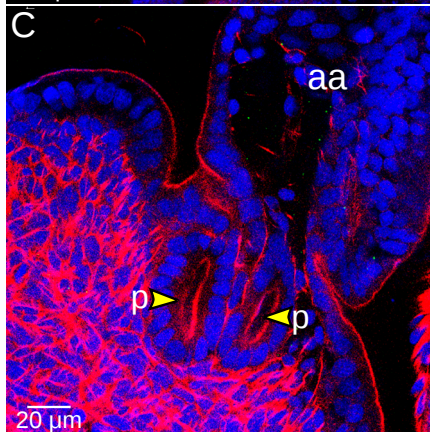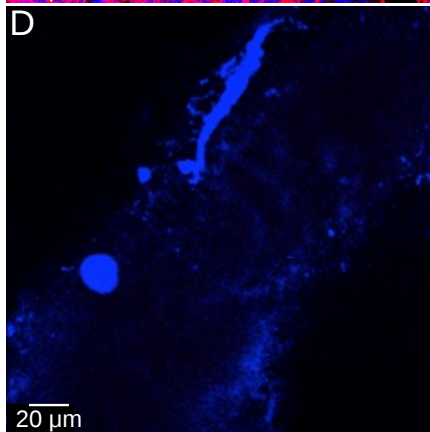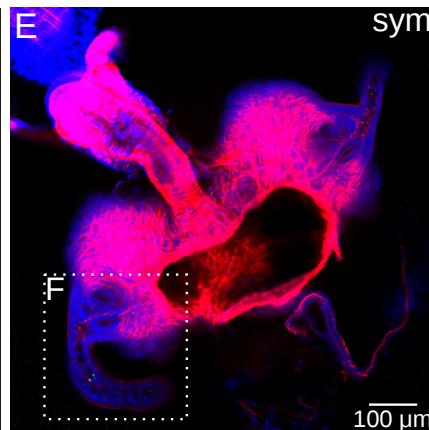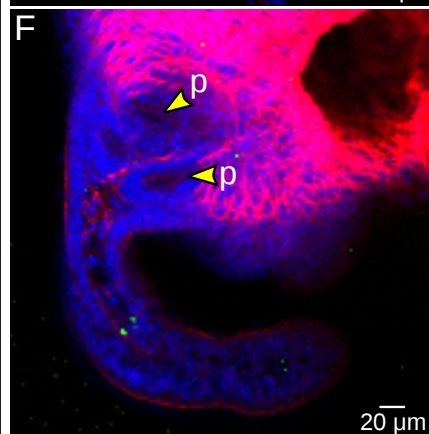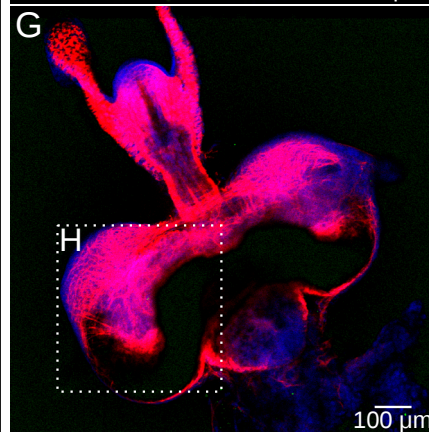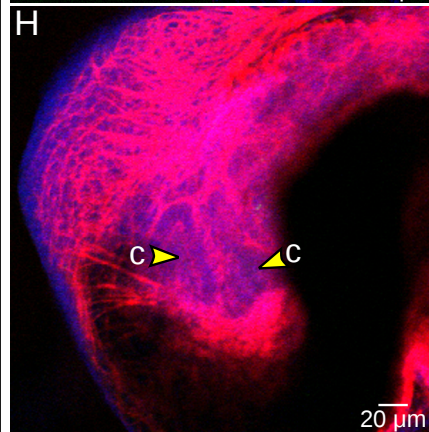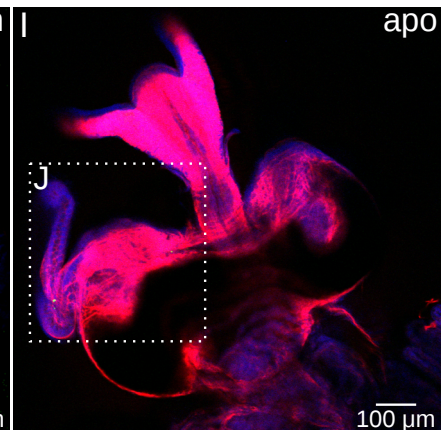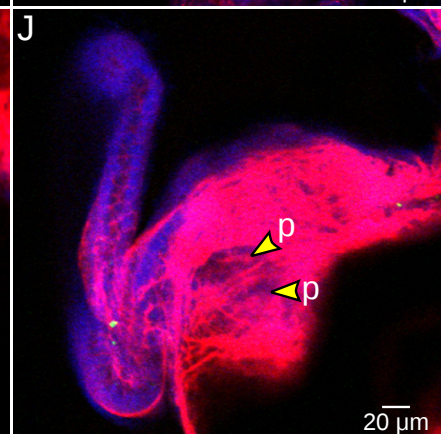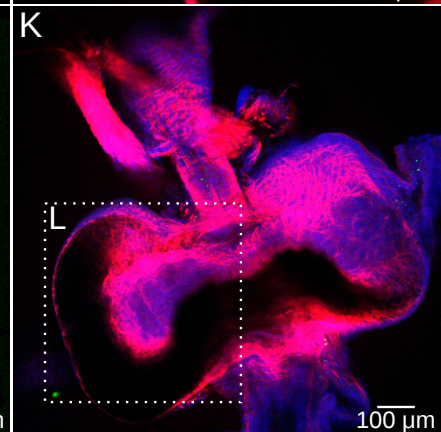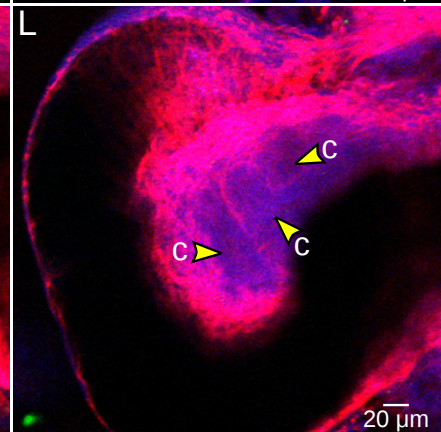

Color Key { Actin  
Nuclei or Mucus (D only)

Supplement: Supplementary file 2 [file MBO3-7-e00632-s002.pdf]

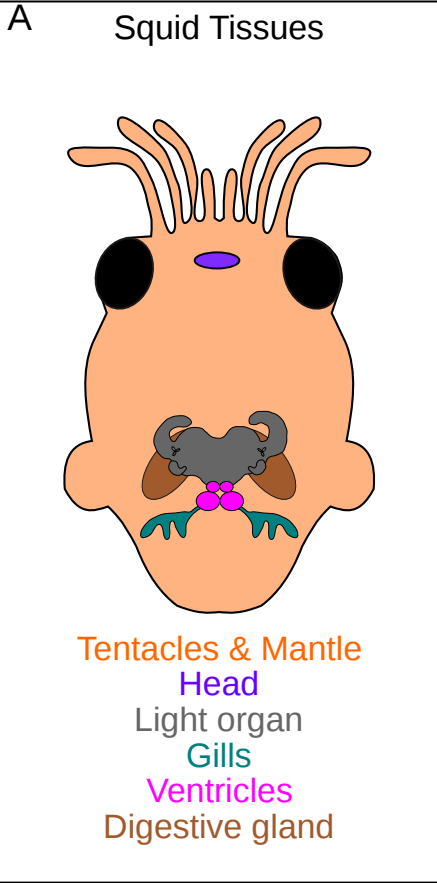

Color Key

Actin  
Nuclei } B-G

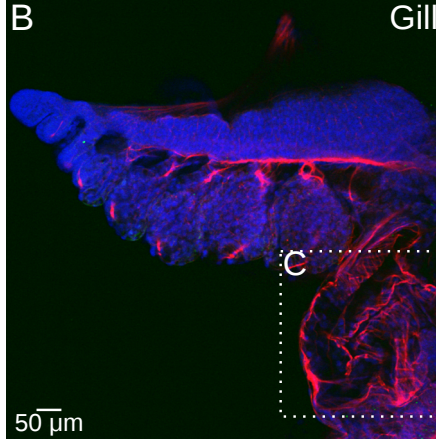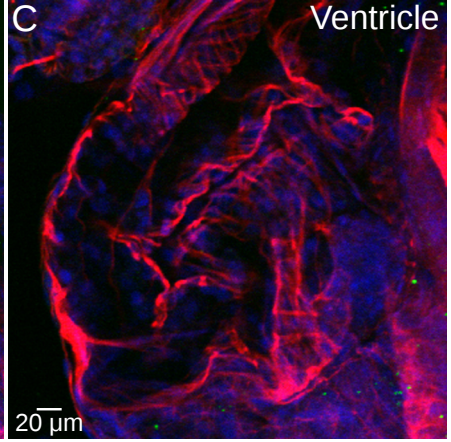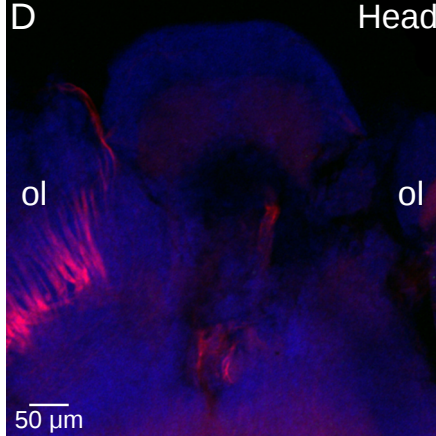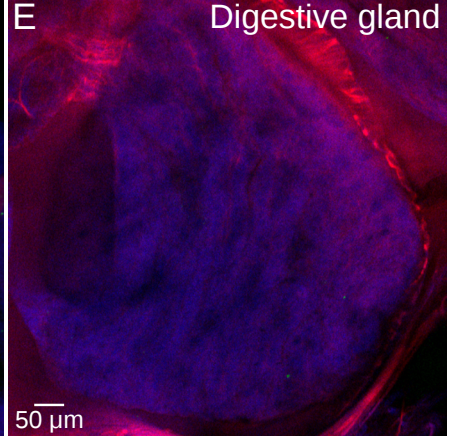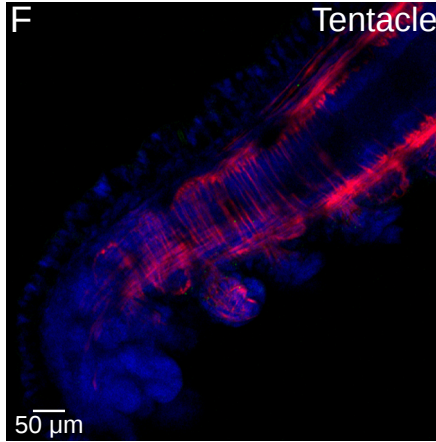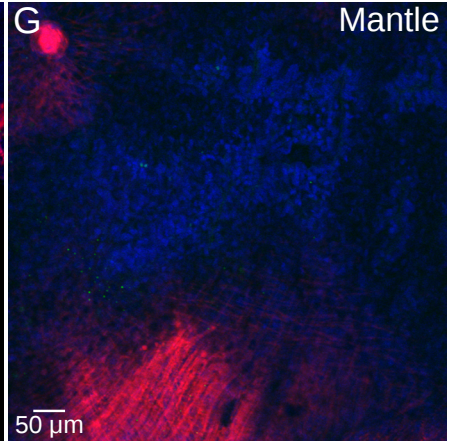

Supplement: Supplementary file 4 [file MBO3-7-e00632-s004.pdf]

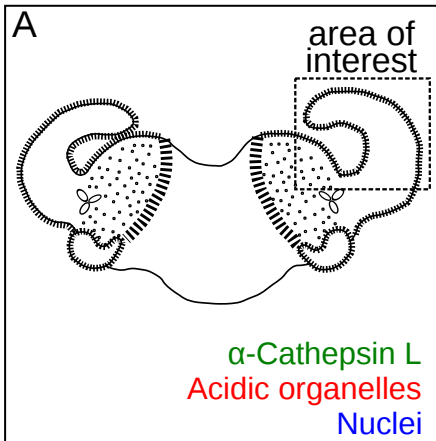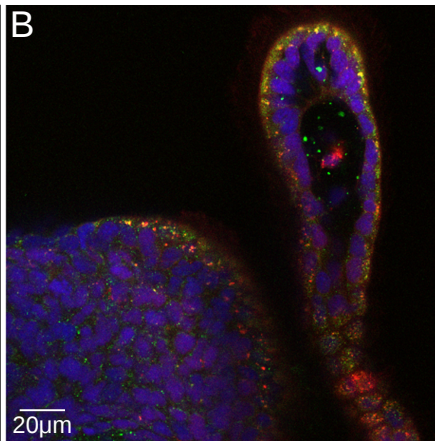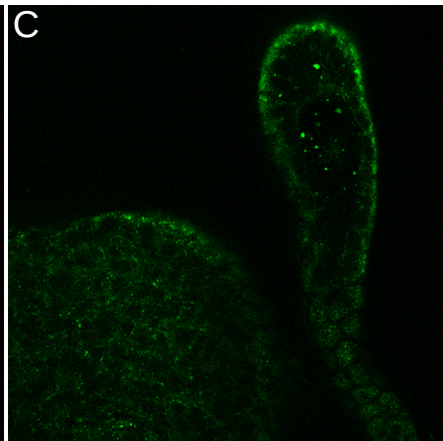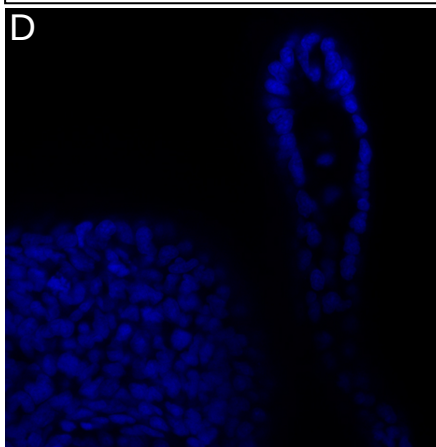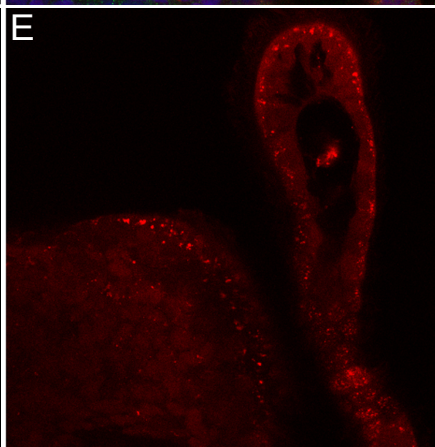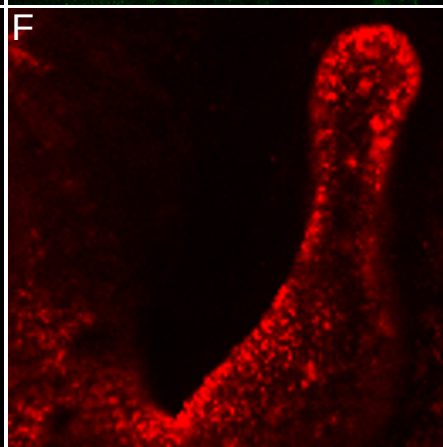

Supplement: Supplementary file 5 [file MBO3-7-e00632-s005.pdf]
