# Supplementary material for: Involvement of a host Cathepsin L in symbiont‐induced cell death
Source: Microbiologyopen. 2018 Apr 24;7(5):e00632. doi: 10.1002/mbo3.632 (PMC6182562; doi:10.1002/mbo3.632)
Supplement: Supplementary file 3 [file MBO3-7-e00632-s003.pdf]

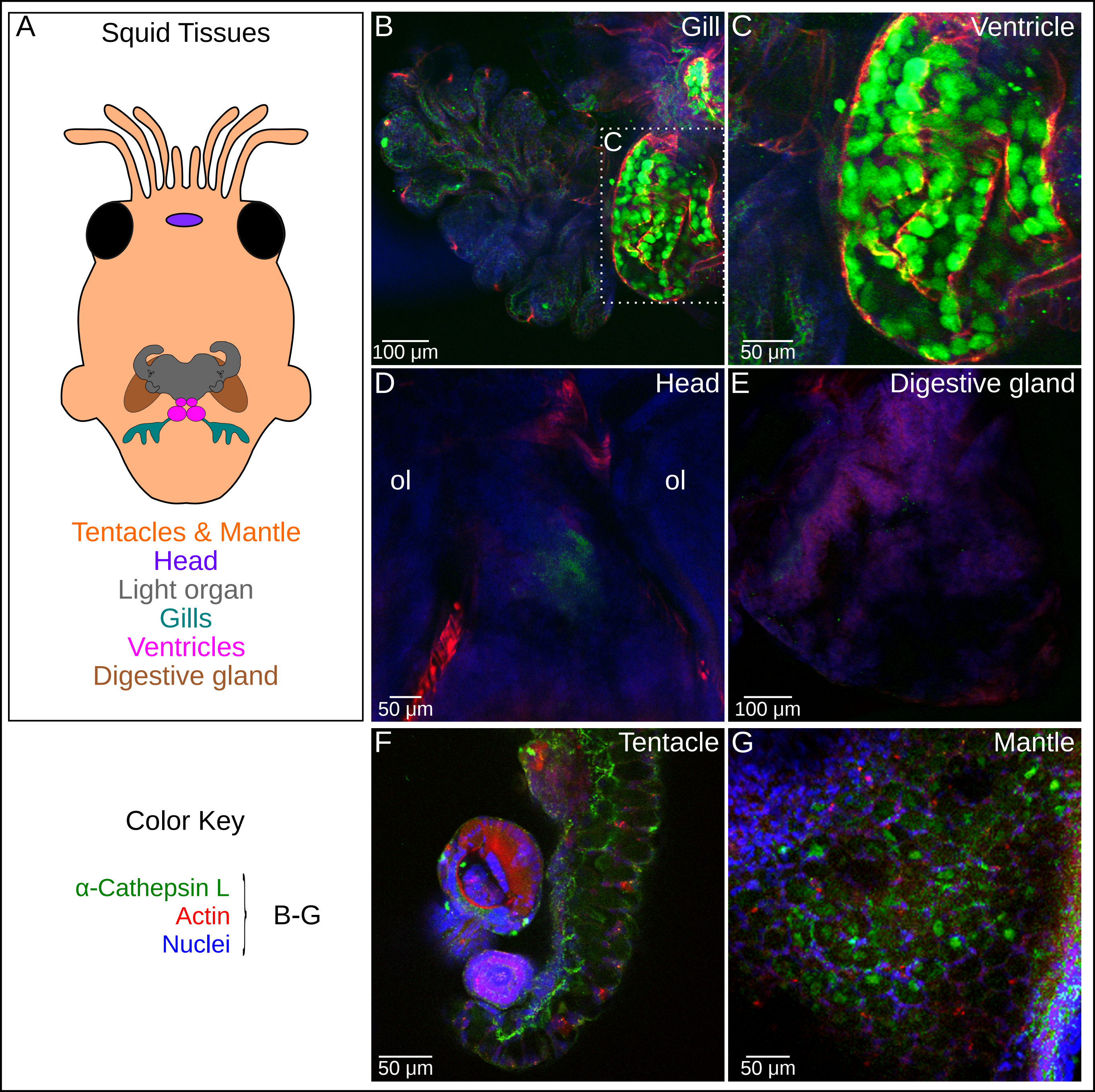
A
Squid Tissues
Tentacles & Mantle
Head
Light organ
Gills
Ventricles
Digestive gland
Color Key
α-Cathepsin L
Actin
Nuclei
B-G
B
Gill
C
100 μm
C
Ventricle
50 μm
D
Head
ol
ol
50 μm
E
Digestive gland
100 μm
F
Tentacle
50 μm
G
Mantle
50 μm
