# Supplementary material for: Involvement of a host Cathepsin L in symbiont‐induced cell death
Source: Microbiologyopen. 2018 Apr 24;7(5):e00632. doi: 10.1002/mbo3.632 (PMC6182562; doi:10.1002/mbo3.632)
Supplement: Supplementary file 6 [file MBO3-7-e00632-s006.pdf]

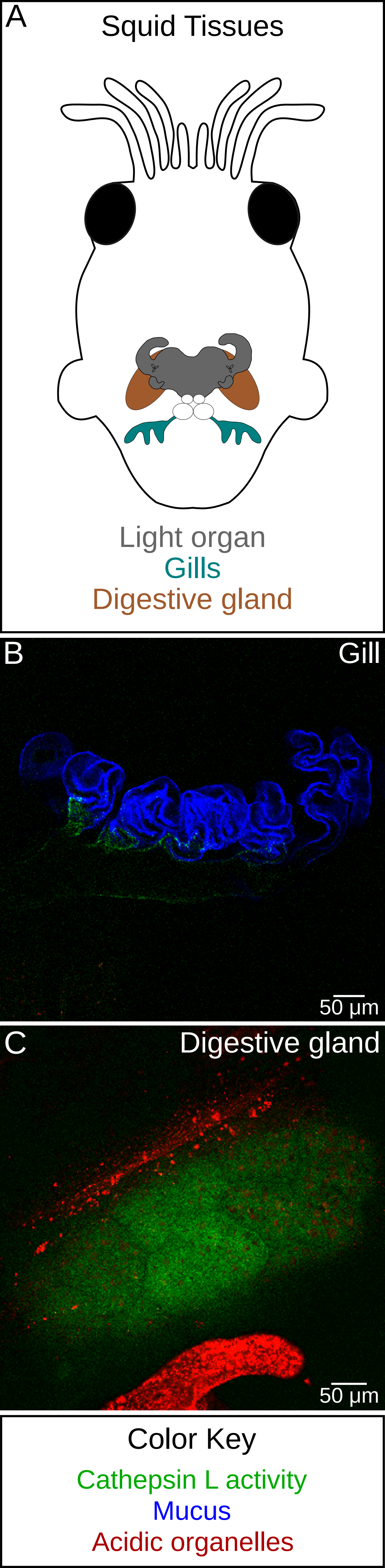

A
Squid Tissues
Light organ
Gills
Digestive gland
B
Gill
50 µm
C
Digestive gland
50 µm
Color Key
Cathepsin L activity
Mucus
Acidic organelles
