# Supplementary material for: Involvement of a host Cathepsin L in symbiont‐induced cell death
Source: Microbiologyopen. 2018 Apr 24;7(5):e00632. doi: 10.1002/mbo3.632 (PMC6182562; doi:10.1002/mbo3.632)
Supplement: Supplementary file 7 [file MBO3-7-e00632-s007.doc]

**Table S1.** Molecular probes used in the localization of *es-cathepsin L* by hybridization chain reaction-fluorescence *in situ* hybridization.

| **Probe #** | **Sequence** |
| --- | --- |
| 1 | AGTGTTGTCCTTCCAGCGATCCAGTTGTAGAGAAAGCCCAACATGATCCG |
| 2 | CGCATTATCCATAAGTCCGCCATTGCAACCCTGATTTCCACAATCATGGG |
| 3 | CTGCCACCTACACCCTTGTAAGGATAATCAGCTTCAGACATAATTCCGCC |
| 4 | GTCAACGTGGCCGGTAACTGTGGCGACAACTTTAGATTTGTCAAAGTGAC |
| 5 | ACCAACAGTTCCTACAGCTTTCTGGAGGGCTTCTTCGCTGCCGGATTTCA |
